# Supplementary material for: Can resistant coral-Symbiodinium associations enable coral communities to survive climate change? A study of a site exposed to long-term hot water input
Source: PeerJ. 2014 Apr 8;2:e327. doi: 10.7717/peerj.327 (PMC3994648; doi:10.7717/peerj.327)
Supplement: Figure S1 [file peerj-02-327-s001.pdf]

ATOLL RESEARCH BULLETIN
NO. 354

REEF ENVIRONMENT AND CORAL FAUNA OF SOUTHERN TAIWAN

BY

CHANG-FENG DAI

ISSUED BY

NATIONAL MUSEUM OF NATURAL HISTORY

SMITHSONIAN INSTITUTION

WASHINGTON D.C., U.S.A.

JULY 1991

REEF ENVIRONMENT AND CORAL FAUNA OF SOUTHERN TAIWAN

BY

CHANG-FENG DAI

ABSTRACT

Southern Taiwan is surrounded by well-developed fringing reefs inhabited by a relatively rich coral fauna. The Taiwanese coral fauna is of considerable biogeographical interest since it borders the region with highest coral diversity. This report presents a description of the reef environment and a revised species checklist of both scleractinian and alcyonacean corals from southern Taiwan. 230 species representing 58 genera of scleractinian corals, 9 species of non-scleractinian reef-building corals, and 40 species of alcyonacean corals were recorded. Among them, 8 genera and 95 species of sclearactinian corals and 28 species of octocorals are new records. With regard to the scleractinian and alcyonacean fauna, southern Taiwan constitutes a part of the Indo-Pacific zoogeographical province and its species diversity is comparable to adjacent areas such as the Ryukyu Islands, Hainan Island, and Guam.

INTRODUCTION

Taiwan borders the region of the West Pacific Ocean with highest coral diversity, which lies between the Philippines and eastern Australia. Because of its central position, and because of the lack of main shallow water areas, Taiwan might be expected to act as a 'stepping stone' in the northward and eastward dispersal of shallow water organisms. A detailed knowledge of the Taiwanese reef fauna and flora will aid in understanding both biogeographical and dispersal questions concerning the western Pacific Ocean.

Although Taiwan lies near the northern latitudinal limit for the development of extensive fringing or barrier reef

---

Institute of Oceanography, National Taiwan University,
P. O. Box 23-13, Taipei, Taiwan, R. O. C.

Manuscript received 28 January 1991; revised 26 April 1991

systems, its sublittoral regions possess coral communities that rival many of those found in lower latitudes in diversity (Jones et al. 1972; Dai, 1988). The coral communities of Taiwan are distributed along the northern, eastern, and southern coasts and are lacking only on the western sandy coast. Other areas supporting coral communities are found at adjacent islands such as Lanyu (Orchid Island), Lutao (Green Island), Hsiao-Liuchiu, Penghu (The Pescadores) and a number of small offshore rocky islets (Fig. 1). Among these localities, southern Taiwan has the highest species diversity of corals.

Most previous work dealing with the Recent coral reefs of southern Taiwan has been in the form of species checklists compiled from scleractinian coral collections. Sugiyama (1937) reports 43 species and 24 genera from a coral collection made by Ehara in southern Taiwan. Kawaguti (1942, 1943) listed 78 species and 34 genera from Garanpi (Oluanpi) and compared the coral fauna there with other collections from Taiwanese waters. He also discussed the geographic distribution of corals along the Taiwanese coast and offshore islands. Later he (Kawaguti, 1953) revised the previous coral collections from Oluanpi and listed 87 species and 35 genera. Some publications by Ma (1957, 1958, 1959) reveal 26 coral species and 18 genera as being collected from Oluanpi. Jones et al. (1972) collected 340 coral specimens from the reefs of southern Taiwan and provided a list of 52 genera and 173 species, of which 121 are new to the study area. Subsequent investigations by Yang et al. (1976, 1977, 1982) increased the number to 245 species and 59 genera. However, most of these works only present a species checklist and a description of the coral fauna but include little systematic and ecological studies. Owing to the confused status of species at that time and the lack of prudent systematic studies, the species lists presented by the previous authors include numerous synonyms. Alcyonacean corals are among the most abundant benthic organisms on the fringing reefs of southern Taiwan, whereas they have rarely been given attention (Jones et al. 1972; Yang, 1985). Utinomi (1959) reported 15 species of alcyonaceans collected from tide-pools of southern Taiwan with the addition of very short descriptions. The species described by him were synonymized by Verseveldt (1980, 1982, 1983) into 11 species.

This report presents a brief description of the reef environment and a revised species checklist of both scleractinian and alcyonacean corals on the fringing reefs in southern Taiwan. Detailed taxonomic descriptions of the coral fauna have been published elsewhere (Dai, 1989, 1991; Hoeksema and Dai, 1991).

REEF ENVIRONMENT

GEOGRAPHY

Taiwan is located on the edge of a continental shelf and, hence, has affinities with continental landforms. To the east, the submarine topography drops steeply to 4000 m, and even to nearly 7000 m in the nearby Ryukyu Trench (Chu, 1971). Tectonically, Taiwan is situated in a continental-arc collision zone between the Asian Plate and the Philippine Sea Plate. The neotectonics of this island during the Holocene has been very evident by its substantial uplift, which may be greater than any other part of the world (Ho, 1975). The average uplift rate of the island has been estimated to range from 4.3 to 5.3 mm/yr (Peng et al. 1977).

The study area, i.e., Hengchun Peninsula, is located at the southern tip of Taiwan (21°55'-22°00'N., 120°40'- 120°52' E.). The peninsula is underlain mainly by Miocene rocks and capped with Pleistocene and Holocene sediments. The average uplift rate of the peninsula since 9000 yr b.p. is 5.3 mm/yr; however, the uplift rate has decreased to 2.5 mm/yr since 2000 yr b.p. (Peng et al. 1977). The high uplift rate of the study area may be partly responsible for the relatively narrow underwater fringing reefs in southern Taiwan. Raised coral reefs are scattered all over the peninsula and they form several levels of coastal terraces fringing the peninsula (Hanzawa, 1931). The prominent emergent reefs of the coastal area are continuations of the presently growing underwater coral reefs (Tsan, 1974).

CLIMATOLOGY

Taiwan is influenced by both continental and marine climates. The island lies partly in the tropics, with the Tropic of Cancer passing through the middle of the island. The tropical climate of southern Taiwan is characterized by marked seasonal variations in temperature and rainfall. Air temperatures are generally high, despite significant seasonal changes, with mean daily air temperature ranging from 20.1 in winter to 28.2 C in summer. Of the average annual rainfall (2200 mm), the majority (77 %) falls in the warmer months (June-September). The extreme seasonal variability of rainfall is evident from the long-term monthly mean rainfall record (Fig. 2). The strong seasonality of rainfall can impart localized seasonal changes in shallow inshore water turbidity and salinity as a consequence of terrigenous runoff.

The predominant winds of southern Taiwan are the seasonal monsoons. Two monsoon seasons are evident in southern

Taiwan, i.e., the northeast winter monsoon (October-April) and the southwest summer monsoon (May-September). Wind velocities are higher in winter and lower in summer with an annual average of 4.1 m/sec (Hsu, 1974). The unusually strong winter monsoon in the Hengchun area (known as "down-hill wind") has a speed of 10-17 m/sec (Hsu, 1974). Since the wind blows from NE to SW, the prevailing swell produced by this winter monsoon is strongest on the west side of Nanwan Bay.

The tropical location of southern Taiwan places it within the zone of typhoon disturbance. On average, the study area suffers direct hits (on typhoon tracks) by 1.17 typhoons per year. The high winds, high seas and generally intense rain accompanying such phenomena can cause considerable changes on reef morphology and coral communities (Stoddart, 1962, 1974; Woodley et al. 1982). Since typhoons that hit southern Taiwan usually blow from SE to NW, the west side of Nanwan Bay is exposed to the catastrophic damage of storms, whereas, the west coast of the Peninsula and the east side of Nanwan Bay are less exposed owing to the shielding by mountains.

OCEANOGRAPHIC SETTING

Several oceanographic survey programs have been conducted in southern Taiwan. Summarized below are data from four major sources: (1) a marine biological data acquisition program pertaining to the construction of a power plant at the west side of Nanwan Bay (Yang et al., 1976, 1977, 1982); (2) ecological surveys on the waters adjacent to the nuclear power plant (Hung et al., 1984); (3) ecological investigations on the waters of Kenting National Park area (Chang and Chen, 1986, 1987); and (4) continuously recorded unpublished data on water temperature and current of Nanwan Bay provided by the Radiation Laboratory of the Taiwan Power Company.

1. Water Temperature

Monthly average sea temperatures at 15 m deep (on reef surface) of southern Taiwan range from 22.5 to 28.2°C (Fig. 3). The variation in sea temperature is seasonal, with its high in summer (July-August) and low in the winter season (December-March), resulting in an annual range of 5-6°C. Differences in daily average sea temperature range from 20.3 to 29.2 °C. Diurnal variation is less than 5°C. Sea temperatures of southern Taiwan are relatively uniform in terms of different localities and depths.

2. Light and Water Turbidity

The average Secchi visibility depth for the reef waters of

Nanwan Bay was higher than 15 m in most areas, except in shallow waters in the northern part of the Bay (Hung et al., 1984). Light attenuation patterns through the water columns of Nanwan Bay (Yang et al. 1982) show that the relative light intensity at depths of 3-5 m varies widely (20-50 %) at different sites and the lowest occurs on the east side of Nanwan Bay. Relative light intensity usually falls below 20% at 20 m deep.

Turbidity caused by river discharge is heaviest on the east coast of the peninsula and at the east side of Nanwan Bay. Yang et al. (1982) reported that the turbidity measured at northeast side of Nanwan Bay three days after a heavy rainfall was 4.3 J.T.U. (Jackson Turbidity Unit), whereas the water turbidity of Nanwan Bay is usually 0.28-1.90 J.T.U.

3. Salinity and Nutrients

Annual variation of salinity measured in Nanwan Bay shows a regular annual pattern; ranging from 32.21-34.97 ppt with its maximum in winter (January-March) and minimum in summer (July-September). The distribution of salinity values is relatively uniform in both winter and summer season (Hung et al. 1984). During most seasons, the salinity is within the optimal range for hermatypic coral growth (34-36 ppt). River discharge has very limited effect on the salinity here.

Dissolved oxygen in the waters of southern Taiwan ranges from 3.77 to 5.10 ml/l. Usually the oxygen contents are highest in winter and lowest in summer. Seawater pH values in Nanwan Bay are relatively homogeneous and range from 7.84 to 8.34. Nutrients concentrations measured in the Bay were: nitrate (NO_3^--N), < 0.05 to 25.01 μM; nitrite (NO_2^--N), < 0.01 to 6.08 μM; phosphate (PO_4^{3-}-P), < 0.03 to 6.42 μM; silicate (-SiO_2^{2-}-Si), < 0.10 to 52.19 μM. BOD of the seawater ranges from 0.00 to 2.33 ± 0.29 ppm. The amount of total lipids ranges from 1.14 to 90.2 mg/l.

Related to concentrations of dissolved nutrients in other coral reef waters (Crossland, 1983), the concentrations of nitrate, nitrite and phosphate in southern Taiwan are unusually high.

4. Tides and Currents

The tidal regime of southern Taiwan is semidiurnal with a cycle of 12 hours and 25 minutes. Spring tides alternate regularly with neap tides. For Nanwan Bay, the mean spring tide range is 1.35 m and the mean neap tide range is 0.63 m. The tidal currents flow from east to west during flood tide and from west to east during ebb tide (Fig. 4). Hourly current recorded at Nanwan Bay shows prominent difference of

current speed between the west and northeast side of the bay (Fan and Yu, 1981). This difference is mainly due to the effect of bottom topography.

The inshore current of the west coast of Hengchun Peninsula flows from SSE to NNW during flood tide and from NNW to SSE during ebb tide with an average speed of 15 cm/sec (ranges from 2 to 53 cm/sec). The current speed on the northwest coast of the peninsula is relatively high indicating its high water energy environment. The relatively fast water flows of southern Taiwan are partly due to the strong Kuroshio current (Fan and Yu, 1981).

MATERIALS AND METHODS

SCLERACTINIAN FAUNA

Scleractinian specimens examined during this study are from three major sources: (1) the collections made by Jones et al. (1972) including a total of 340 coral specimens which are deposited at the Institute of Oceanography, National Taiwan University (TUIO), Taipei; (2) the collections made by Yang et al. (1976-1982) including about 1000 specimens and also deposited at TUIO, and (3) personal collections of the author made during several dives in 1981-1984 and 1985-87, with a total of about 500 specimens, of which representatives of species have been deposited at the Peabody Museum of Natural History, Yale University, New Haven, Connecticut, USA. Collections were made at sites throughout the fringing reefs of southern Taiwan (Fig. 1). The identification of the scleractinian species was carried out largely following the species concepts proposed by Veron and his co-workers (Veron and Pichon, 1976, 1979, 1982; Veron et al. 1977; Veron and Wallace, 1984; Hoeksema, 1989).

ALCYONACEAN FAUNA

Alcyonacean corals were also collected at stations as shown in Fig. 1. Whole colonies were collected if size permitted and otherwise a longitudinal slice (including capitula, stalk, and holdfast) was collected. Morphological characters of living colonies were recorded in situ and in addition underwater photos were taken. Specimens were placed in plastic bags labeled with codes corresponding to photographic tags.

The external morphology of polyps was examined with a dissecting microscope. Since different parts of an alcyonacean colony often contain different types of sclerites, four

sclerite preparations of a colony are prepared for identification, namely (1) surface layer of the capitulum or lobe, (2) interior part of the capitulum or lobe, (3) surface layer of the stalk, and (4) interior of the stalk. Sclerites were isolated by using a 20 % NaOH solution to disintegrate the tissues.

A total of about 220 alcyonacean specimens was examined. Species identifications were made using information published in Verseveldt (1980, 1982, 1983), Verseveldt and Alderslade (1982), Bayer (1981), and references cited therein. The specimens are deposited at the following institutions: (1) Peabody Museum of Natural History, Yale University, New Haven, USA; (2) Institute of Oceanography, Taiwan University, Taipei; and (3) Academia Sinica, Taipei, Taiwan.

RESULTS

SCLERACTINIA FAUNA

Table 1 presents a preliminary species list of scleractinian and non-scleractinian reef-building corals from the reefs of southern Taiwan. A review of previous records and the bathymetric distribution of each species are also given. A few species and genera listed by previous authors are omitted here; these have been reassigned to other taxa or have been synonymized.

In the present study, 230 species are recognized, representing 58 genera of scleractinian corals. Nine species of non-scleractinian reef-building corals including Tubipora musica, Heliopora coerulea and seven Millepora species are also listed here. Among them, 8 genera (Plerogyra, Physogyra, Gardineroseris, Pectinia, Blastomussa, Caulastrea and Oxypora), and 95 species of sclearactinian corals are new records. Most of the species assignments correspond to those by Veron and Pichon (1976, 1980, 1982), Veron et al. (1977), Veron and Wallace (1984), and Hoeksema (1989) for widespread species. Since the collecting effort in southern Taiwan is not very intense, there are likely to be some undiscovered scleractinian such as Madracis, Physophyllia, Acrhelia, and Cynarina. Additional species of particular genera such as Acropora, Montipora, and Porites, are also likely to be discovered since the traditional taxonomic difficulties related to these genera indicate that species may easily be overlooked. New species from deep water may similarly exist since depths greater than 35 m have rarely been studied in the study area. However, the majority of species found in southern Taiwan is included in Table 1.

ALCYONACEAN FAUNA

Table 2 lists the known shallow water alcyonacean species on the reefs of southern Taiwan. The list includes 40 species of which 28 are new records. Eight species do not conform to previous descriptions and remain unassigned.

Alcyonacean corals in southern Taiwan are mainly distributed in the areas around the two southernmost tips. The most extensive and abundant alcyonacean community was found at the west side of Nanwan Bay. This area is exposed to moderately strong currents and occasional storm surges. Alcyonaceans were rarely found on the protected reefs such as the east side of Nanwan Bay.

Although some species are common in tidal pools, alcyonaceans rarely appear in very shallow water (0-2 m) possibly due to their lack of a strong skeleton. However, they become common on deeper flat reef surfaces down to 5 m in depth. In this region, the soft corals are represented by large encrusting alcyonaceans such as *Sinularia exilis*, *S. facile*, and *Lobophytum pauciflorum*. Colonies of these species often reach 2 m in diameter or more. Alcyonaceans are abundant in intermediate waters of exposed reefs such as the submarine terrace and the reef front and occur in various shapes and colors. The most abundant species are: *Sarcophyton trochel-iophorum*, *S. crassocaule*, and *Lobophytum sarcophytoides*. They usually form large stands of mushroom-like, bush-like or basin-like colonies. In deep waters, alcyonaceans are represented by small colonies of *Sarcophyton* spp. and *Sinularia* spp.

DISCUSSION

SCLERACTINIA FAUNA

With 61 genera and 235 species, the scleractinian fauna of southern Taiwan is very rich and is comparable to the richest areas in the west Pacific in terms of species diversity. Veron (1985) includes Taiwan in the 60 genera contour on the map of worldwide coral distributions. This study confirms his estimation, however, a significant decrease of species diversity from south to north on the island can be expected. Probably only southern Taiwan has such high species diversity.

Despite this high diversity, there are few coral species endemic for southern Taiwan, except *Fungia* (*Pleuractis*) *taiwanensis* (Hoeksema and Dai, 1991). The apparent small

degree of endemism is related to the fact that the fauna is of relatively recent origin and to the lack of geographical isolation of the island. By using radiocarbon methods, coral rocks collected from the seashore (1-2 m above sea level) around Hengchun Peninsula have been dated as 1300-1500 yr b.p. (Peng et al. 1977). These data indicate that the living reefs of southern Taiwan may be younger than 1500 years. The corals of southern Taiwan represent a newly colonized fauna; the prevailing northward current passing Taiwan, the Kuroshio Current, is responsible for colonization from the south. Southern Taiwan is located about 200 miles north of Luzon, and there is an additional series of small islets lying in between them. The Kuroshio current is a continuation of the North Equatorial Current which flows north from the central Philippines toward Taiwan at an average velocity of 1.0 knot (Nitani, 1972). Drift time from the Philippines to southern Taiwan is thus about eight days. Since coral larvae may remain in the plankton for a few weeks (Fadlallah, 1983; Richmond and Hunter, 1990), larvae from coral reefs of the Philippines and central Pacific Ocean can reach the waters of southern Taiwan and still have the ability to settle. Because of its close position to the Indo-Pacific scleractinian diversity center and because of its favorable environment, southern Taiwan thus has a very rich coral fauna despite its small reef area.

In addition, the scleractinian fauna in southern Taiwan may have a high species turnover rate. Catastrophic events such as typhoons and heavy sedimentation could be occurring frequently enough to cause local extinctions. Indeed, in spite of the fact that a large number of species was discovered during this study, other species reported by previous authors in the study area were not observed even under intense searching. The high recruitment rates with frequent disturbances seem to be the important factors in maintaining the species diversity of this fauna.

205 species (89%) recorded from southern Taiwan also occur on the Great Barrier Reef. Most of the species common to both regions do not show significant taxonomic differences. Similarly, Veron (1986) reported that 89% of the scleractinians recorded from Ishigaki I. also occur on the Great Barrier Reef. This indicates that the distribution of Indo-Pacific corals is generally homogeneous throughout the province at both generic and specific levels (Wells, 1969; Veron, 1985).

ALCYONACEAN FAUNA

The alcyonacean fauna of the study area is a part of the Indo-Pacific zoogeographical province. Most of the species

reported here are widely distributed in the province. The species diversity of alcyonacean fauna in southern Taiwan is comparable to the Ryukyu Islands (36 species; Utinomi, 1976, 1977a, b) and Hainan Island (18 species; Li, 1982). However, the major species reported in this study represent a relatively small proportion of species considered valid by Verseveldt (1980, 1982, 1983). For example, only 17% of Sinularia and Sarcophyton species and 13% of Lobophytum species are included in the alcyonacean species list.

The type locality of Asterospicularia laurae is in southern Taiwan (Utinomi, 1951) where it seems to have a restricted distribution, mainly in tide pools. The Xeniidae have been reported dominant in some reef environments in the Red Sea (Benayahu, 1985) and the central Great Barrier Reef (Dinesen, 1983), but they are relatively rare on the reefs of southern Taiwan. Several genera which have been reported abundant on some Indo-Pacific reefs, such as Litophyton, Lemnalia, Efflatounaria, and Capnella have not been recorded in the study area. Due to the systematic complexity of alcyonacean corals, their Indo-Pacific fauna is largely undescribed and hence it is premature to discuss their biogeography.

ACKNOWLEDGEMENTS

I am grateful to Dr. Bert W. Hoeksema for his efforts on taxonomy of Taiwanese mushroom corals and for his critical reading of the manuscript. This study was supported by the Kenting National Park Headquarters, Bureau of Construction, Ministry of Interior, R. O. C.

REFERENCES

Bayer, F. H. 1981. Key to the genera of Octocorallia exclusive of Pennatulacea (Coelenterata: Anthozoa), with diagnosis of new taxa. Proc. Biol. Soc. Wash. 94:902-947.

Benayahu, Y. 1985. Faunistic composition and patterns in the distribution of soft corals (Octocorallia: Alcyonacea) along the coral reefs of Sinai Peninsula. Proc. 5th Internat. Coral Reef Cong., Tahiti 6:255-260.

Chang, K. H. and Y. M. Chen. 1986. Nutrients in coral reef waters of the Kenting National Park area. Conservation Research Report No. 34-3, 102 pp. The Kenting National Park Headquarters. (in Chinese).

Chang, K. H. and Y. M. Chen. 1987. Nutrients in coral reef waters of the Kenting National Park area II. Conserva-

tion Research Report No. 42-1, 61 pp. The Kenting National Park Headquarters. (in Chinese).
Chu, T. Y. 1971. Environmental study of the surrounding waters of Taiwan. Sino-American Science Cooperation Colloquium on Ocean Resources. Preprints Vol. II.
Crossland, C. J. 1983. Dissolved nutrients in coral reef waters. Pages 56-68, In: Barnes, D. J. (ed.), Perspec tives on Coral Reefs, Australian Institute of Marine Science.
Dai, C. F. 1988. Coral communities of southern Taiwan. Proc. 6th Int. Nat. Coral Reef Symp. 2:647-652.
Dai, C. F. 1989. Scleractinia of Taiwan. I. Families Astrocoeniidae and Pocilloporidae. Acta Oceanogr. Taiwanica 22:83-101.
Dai, C. F. 1991. Scleractinia of Taiwan. III. Family Agariciidae. Acta Oceanogr. Taiwanica (in press).
Dinesen, Z. D. 1983. Patterns in the distribution of soft corals across the central Great Barrier Reef. Coral Reefs 1:229-236.
Fadlallah, Y. H. 1983. Sexual reproduction, development and larval biology in Scleractinian corals. Coral Reefs 2:129-150.
Fan, K. L. and C. Y. Yu. 1981. A study of water masses in the seas of southernmost Taiwan. Acta Oceanographica Taiwanica 12:94-111.
Hanzawa, S. 1931. Notes on the raised coral reefs and their equivalent deposits in Taiwan (Formosa) and adjacent islets. Rec. Oceanog. Wk. Jap. 3:37-52, pls. 1-10.
Ho, C. S. 1975. An introduction to the geology of Taiwan. Min. Eco. Affairs, R.O.C. 153 pp.
Hoeksema, B. W. 1989. Taxonomy, phylogeny and biogeography of mushroom corals (scleractinia: Fungiidae). Zool. Verh. Leiden 254:1-295.
Hoeksema, B. W. and C. F. Dai. 1991. Scleractinia of Taiwan. II. Family Fungiidae (including a new species). Bull. Inst. Zool. Academia Sinica 30 (in press).
Hsu, S.-H. 1974. Weather analysis at Hengchun of Taiwan. Sci. Rep. Pingtung Agriculture College 14:237-256. (in Chinese).
Hung, T. C., C. C. Su, Y. M. Chiang, T. H. Tan, K. H. Chang, R. T. Yang, Y. M. Cheng, K. L. Fan and H. T. Chang. 1984. An ecological survey on the waters adjacent to the nuclear power plant in southern Taiwan. V. The progress report of the fifth year study (1983-1984) and the summary report of five years preoperational stage studies (1979-1984). Nat. Sci. Comm. Prob. Environ., Spec. Publ. No. 27, 214 pp.
Jones, O. A., R. H. Randall, Y. M. Cheng, H. T. Kami and S. M. Mak. 1972. A marine biological survey of southern Taiwan with emphasis on corals and fishes. Inst. Oceanogr., Nat. Taiwan Univ. Spec. Publ. 1, 93 p.
Kawaguti, S. 1942. Coral fauna of the Taiwan waters. Kagaku

no Taiwan 11:1-6.
Kawaguti, S. 1943. Coral fauna of Garampi. Trans. Formosan Nat. Hist. Soc. 33:258-259.
Kawaguti, S. 1953. Coral fauna of the island of Botel Tobago, Formosa with a list of corals from the Formosan waters. Biol. J. Okoyama Univ. 1:185-198.
Li, C.-P. 1982. Studies of the Alcyonacea of the South China Sea. I. Alcyonacea from Yalong Bay. Tropic Oceanology 1:156-169.
Ma, T. Y. H. 1957. The effect of warm and cold currents in the southern-western Pacific on the growth rate of reef corals. Oceanographia Sinica 5:1-34.
Ma, T. Y. H. 1958. The relation of growth rate of reef corals to surface temperature of sea water as basis for study of causes of diastrophisms instigating evolution of life. Research on the part climate and continental drift. Vol. 14, World Book Co., Taipei.
Ma, T. Y. H. 1959. Effect of water temperature on growth rate of corals. Oceanographia Sinica, Spec. Vol. 1, 116 pp. Nitani, H. 1972. Beginning of Kuroshio. p. 129-164, In: Stommel, H. and K. Yoshida (eds.), Kuroshio: Physical aspects of the Japan Current. Univ. Washington Press, Seattle, Washington, USA.
Peng, T. H., Y. H. Li, and F. T. Wu. 1977. Tectonic uplift rates of the Taiwan island since the early Holocene. Mem. Geol. Soc. China 2:57-69.
Randall, R. H. and Y. M. Cheng. 1984. Recent corals of Taiwan. Part III. Shallow water hydrozoan corals. Acta Geologica Taiwanica 22:35-99.
Richmond, R. H. and C. L. Hunter. 1990. Reproduction and recruitment of corals: comparisons among the Caribbean, the tropical Pacific, and the Red Sea. Mar. Ecol. Prog. Ser. 60:185-203.
Stoddart, D. R. 1962. Catastrophic storm effects on the British Honduras Reefs and Cays. Nature 196:512-515.
Stoddart, D. R. 1974. Post-hurricane changes on the British Honduras reefs: resurvey of 1972. Proc. 2nd Internat. Coral Reef Symp. 2:473-483.
Sugiyama, T. 1937. Reef building corals of Japanese coast. Pap. Inst. Geol. Paleont., Tohoku Imp. Univ. 26:1-60.
Tsan, S. F. 1974. Stratigraphy and structure of the Hengchun Peninsula, with special reference to a Miocene Olistostrome. Bull. Geol. Survey Taiwan 24:99-108, pl. 1.
Utinomi, H. 1951. Asterospicularia laurae, n. gen. n. sp., the type of a new family of alcyonarians with stellate spicules. Pac. Sci. 5(2):190-196.
Utinomi, H. 1959. Fleshy alcyonarians from southern Formosa. Publ. Seto Mar. Biol. Lab., 7(3):303-312.
Utinomi, H. 1976. Shallow-water octocorals of the Ryukyu Archipelago (part I.). Sesoko Mar. Sci. Lab. Tech. Rep. 4:1-5.
Utinomi, H. 1977a. Shallow-water octocorals of the Ryukyu

Archipelago (part II). Sesoko Mar. Sci. Lab. Tech. Rep. 5:1-11.

Utinomi, H. 1977b. Shallow-water octocorals of the Ryukyu Archipelago (part III). Sesoko Mar. Sci. Lab. Tech. Rep. 5:13-14, pls. 1-6.

Veron, J. E. N. 1985. Aspects of the biogeography of hermatypic corals. Proc. 5th Internat. Coral Congr. 4:83-88.

Veron, J. E. N. 1986. Distribution of reef-building corals. Oceanus 29:27-31.

Veron, J. E. N. and M. Pichon. 1976. Scleractinia of Eastern Australia. Part I. Families Thamnasteriidae, Astrocoeniidae, Pocilloporidae. Aust. Inst. Mar. Sci. Monogr. Ser. 1, 86 pages.

Veron, J. E. N. and M. Pichon. 1982. Scleractinia of Eastern Australia. Part VI. Family Poritidae. Aust. Inst. Mar. Sci., Monogr. Ser. 5, 159 pages.

Veron, J. E. N. and C. C. Wallace. 1984. Scleractinia of Eastern Australia. Part V. Family Acroporidae. Aust. Inst. Mar. Sci., Monogr. Ser. 6, 503 pages.

Veron, J. E. N., M. Pichon and M. Wijsman-Best. 1977. Scleractinia of Eastern Australia. Part II. Families Faviidae, Trachylliidae. Aust. Inst. Mar. Sci., Monogr. Ser. 3, 233 pages.

Verseveldt, J. 1974. Octocorallia from New Caledonia. Zool. Meded. 48:95-112, fig. 1-17, pl. 1-5.

Verseveldt, J. 1980. A revision of the genus Sinularia May (Octocorallia, Alcyonacea). Zool. Verh. Leiden 179:1-128, pls. 1-38.

Verseveldt, J. 1982. A revision of the genus Sarcophyton Lesson (Octocorallia, Alcyonacea). Zool. Verh. Leiden 192:1-91, pls. 1-24.

Verseveldt, J. 1983. A revision of the genus Lobophytum von Marenzeller (Octocorallia, Alcyonacea). Zool. Verh. Leiden 200:1-103, pls. 1-31.

Verseveldt, J. and P. Alderslade. 1982. Descriptions of types and other alcyonacean material (Coelenterata: Octocorallia) in the Australian Museum. Rec. Aust. Mus. 34:619-647, 8 pls.

Wells, J. W. 1969. Aspects of Pacific coral reefs. Micronesica 5:317-322.

Woodley, J. D. et al. 1981. Hurricane Allen's impact on Jamaican coral reefs. Science 214:749-755.

Yabe, H. and T. Sugiyama. 1935. Geological and geographical distribution of reef-corals in Japan. J. Paleont. 9:183-217.

Yabe, H. and T. Sugiyama. 1936. Revised list of the reef corals from the Japanese seas and of the fossil reef corals of the raised reefs and the Ryukyu limestone of Japan. J. Geol. Soc. Japan pp. 379-403.

Yabe H. and T. Sugiyama. 1941. Recent reef-building corals from Japan and the South Sea Islands under the Japanese Mandate. Sci. Rep. Tohoku Univ. 2nd Ser. (Geol.)

Special vol. II. P. 67-91, pl. 60-104.

Yang, R. T. 1985. Coral communities in Nan-wan Bay (Taiwan). Proc. 5th Internat. Coral Reef Congr., Tahiti 6:273-278.

Yang, R. T., C. C. Huang, C. H. Wang, S. Z. Yeh, Y. F. Jan, S. L. Liu, C. H. Chen, S. J. Chen, L. F. Chang, C. L. Sun, W. Huang and T. F. Tsai. 1976. A marine biological data acquisition program pertaining to the construction of a power plant in the Nan-wan Bay area. Phase I. A preliminary reconnaissance survey. Inst. Oceanogr., Nat. Taiwan Univ. Special Publ. 11, 134 p.

Yang, R. T., C. C. Huang, C. H. Wang, S. Z. Yeh, Y. F. Jan, S. J. Liu, C. H. Chen, S. J. Chen, L. F. Chang and C. F. Tsai. 1977. A marine biological data acquisition program pertaining to the construction of a power plant in the Nan-wan Bay area. Phase II. Biological data acquisition. Inst. Oceanogr., Nat. Taiwan Univ., Spec. Publ. 11, pp. 1-314.

Yang, R. T., C. F. Dai, S. Z. Yeh, C. L. Sun, F. Y. Su, S. Y. Liao, Y. K. Hsu, C. L. Chang, and T. Y. Chou. 1982. Ecology and distribution of coral communities in Nanwan Bay, Taiwan. Spec. Publ. 40, 74 pp.

Table 1. The known scleractinian and non-scleractinian reef-building corals on the fringing reefs of Southern Taiwan. Key to previous records, S: Yabe and Sugiyama (1935, 1936, 1941), Sugiyama (1937); K: Kawaguti (1942, 1943, 1953); M: Ma (1957, 1958, 1959); J: Jones *et al.* (1972); Y: Yang *et al.* (1976, 1977, 1982); R: Randall and Cheng (1984); H: Hoeksema and Dai (1991); N: synonymized from the above records. Distribution of coral species is based on collection records and field observation records from the following depths: A: recorded from 0-5 m; B: recorded from 5-15 m; C: recorded from 15-25 m. '*' indicates species not examined in this study because specimens were lost or not available.

Species	Previous record	Depth A	Depth B	Depth C
CLASS ANTHOZOA				
SUBCLASS ZOANTHARIA				
ORDER SCLERACTINIA				
Family ASTROCOENIIDAE				
Stylocoeniella armata Ehrenberg	J,Y	+	+	
S. quentheri Bassett-Smith	J,Y	+	+	
Family THAMNASTERIIDAE				
Psammocora profundacellar Gardiner	K,Y	+		
P. digitata Edwards & Haime	N	+	+	
P. contigua (Esper)	K,Y	+	+	
P. brighami Vaughan *	K			
P. verrilli Vaughan *	K			
Famliy POCILLOPORIDAE				
Pocillopora damicornis Linnaeus	S,K,J,Y	+	+	
P. eydouxi Edwards & Haime	K,J,Y	+	+	+
P. meandrina (Dana)	S,K,J,Y	+	+	
P. verrucosa Ellis & Solander	K,J,Y	+	+	+
P. woodjonesi Vaughan		+	+	
Seriatopora caliendrum Ehrenberg	K,J,Y	+	+	
S. hystrix Dana	K,Y	+	+	
Stylophora pistillata (Esper)	K,J,Y			
Family ACROPORIDAE				
Acropora palifera (Lamarck)	K,J,Y	+		
A. cuneata (Dana)		+		
A. humilis (Dana)	K,J,Y	+	+	
A. gemmifera (Brook)		+		
A. monticulosa (Brugemann)	J,Y	+	+	
A. digitifera (Dana)	J,Y	+	+	+
A. glauca (Brook)	N	+	+	
A. robusta (Dana)	N	+	+	
A. palmerae Wells	J,Y	+		

Table 1. (continued).

Species	Previous record	Depth A	B	C
A. nobilis (Dana)	N	+	+	
A. grandis (Brook)	N	+	+	
A. formosa (Dana)	S,K,J,Y	+	+	+
A. valenciennesi (Edwards & Haime)	N		+	+
A. microphthalma (Verrill)	N	+	+	
A. aspera (Dana)	N	+	+	+
A. pulchra (Brook)	N		+	
A. millepora (Ehrenberg)	N	+	+	
A. tenuis (Dana)	Y	+	+	
A. dendrum (Basett-Smith)		+	+	
A. selago (Studer)	N		+	+
A. cytherea (Dana)	N	+	+	+
A. microclados (Ehrenberg)			+	
A. hyacinthus (Dana)	K,Y	+	+	
A. anthocercis (Brook)		+	+	
A. latistella (Brook)		+	+	
A. studeri (Brook)	S,K,Y	+	+	+
A. nana (Studer)	J,Y	+	+	+
A. azurea Veron and Wallace		+	+	
A. cerealis (Dana)	K,J,Y	+	+	+
A. nasuta (Dana)	J,Y	+	+	+
A. secale (Studer)			+	+
A. valida (Dana)	J,Y	+	+	+
A. clathrata (Brook)	N		+	
A. divaricata (Dana)		+	+	
A. florida (Dana)		+	+	
A. austera (Dana)			+	+
A. granulosa (Edwards & Haime)			+	+
A. acuminata Verrill	J,Y	+	+	
A. angulata (Quelch) *	K,Y			
A. sp 1		+	+	
A. sp 2		+		
Anacropora matthaii Pillai			+	
Astreopora myriophthalma (Lamarck)	J,Y	+	+	
A. listeri Bernard		+	+	
A. gracilis Bernard			+	
A. randalli Lamberts		+	+	
A. cucullata Lamberts			+	
A. suggesta Wells *	J,Y		+	
Montipora monasteriata (Forskal)	N	+	+	+
M. tuberculosa (Lamarck)			+	+
M. peltiformis Bernard	N	+	+	
M. turgescens Bernard		+		
M. spumosa (Lamarck)		+	+	
M. undata Bernard			+	+

Table 1. (continued).

Species	Previous record	Depth A	Depth B	Depth C
M. verrucosa (Lamarck)	K,J,Y	+	+	+
M. danae Edwards & Haime		+	+	
M. foveolata (Dana)	K,J,Y	+	+	
M. venosa (Ehrenberg)	J,Y	+		
M. angulata (Lamarck)			+	
M. digitata (Dana)	N		+	+
M. hispida (Dana)	J,Y	+	+	
M. efflorescens Bernard		+	+	
M. nodosa (Dana)		+	+	
M. grisea Bernard		+		
M. stellata Bernard	N	+	+	+
M. informis Bernard	K,J,Y	+	+	+
M. foliosa (Pallas)	S,M,K,J,Y	+	+	+
M. aequituberculata Bernard	N		+	+
M. incrassata (Dana)	N	+	+	
M. ehrenbergi Bernard	Y	+	+	
M. marshallensis Wells	J,Y	+	+	
M. lichen Dana	J,Y	+		
M. edwardsi Bernard	K,J,Y	+	+	
M. sp. 1		+	+	
M. sp. 2		+	+	
Family AGARICIIDAE				
Pavona clavus (Dana)	M,J,Y	+	+	+
P. divaricata Lamarck	K,Y		+	+
P. decussata Dana			+	+
P. cactus (Forskal)			+	+
P. maldivensis (Gardiner)	M,K,Y		+	
P. varians Verrill	J,Y	+	+	+
P. venosa (Ehrenberg)	J,Y	+	+	+
P. frondifera (Lamarck) *	S,K			
P. lilacea (Klunzinger) *	S,K			
P. gardineri Van der Horst *	S,K			
Gardineroseris planulata (Dana)	N	+	+	
Leptoseris hawaiiensis Vaughan	J,Y		+	+
L. incrustans (Quelch)	J,Y		+	+
L. explanata Yabe & Sugiyama			+	+
L. yabei (Pillai & Scheer)			+	+
L. tenuis Van der Horst				+
Coeloseris mayeri Vaughan	M,K,J,Y	+	+	
Pachyseris rugosa (Lamarck)	J,Y	+	+	+
P. speciosa (Dana)	K,J,Y		+	+

Table 1. (continued).

Species	Previous record	Depth A	Depth B	Depth C
**Family SIDERASTREIDAE**				
Coscinarea columna (Dana)	J,Y	+	+	
**Family FUNGIIDAE**				
Fungia (Cycloseris) sinensis (E. & H.)	H		+	+
F. (C.) cyclolites Lamarck	H		+	+
F. (C.) fragilis (Alcock)	H			+
F. (C.) costulata Ortmann	H			+
F. (C.) vaughani Boschma	H		+	+
F. (Verrillofungia) repanda Dana	H		+	+
F. (V.) concinna Verrill	H		+	+
F. (Danafungia) horrida Dana	H		+	+
F. (D.) scruposa Klunzinger	H		+	+
F. (Fungia) fungites (Linnaeus)	H		+	+
F. (Wellsofungia) granulosa Klünzinger	H			+
F. (Pleuractis) moluccensis V. d. Horst	H		+	
F. (P.) taiwanensis Hoeksema & Dai	H		+	
F. (P.) gravis Nemenzo	H		+	
F. (P.) paumotensis Stutchbury	H		+	+
F. (Lobactis) scutaria Lamarck	H		+	+
Ctenactis echinata (Pallas)	H			+
Herpolitha limax (Esper)	H		+	+
Polyphyllia talpina (Lamarck)	H		+	+
Sandalolitha robusta (Quelch)	H		+	+
S. dentata Quelch	H		+	+
Lithophyllon undulatum (Rehberg)	H	+	+	+
L. mokai Hoeksema	H		+	+
Podabacia crustacea (Pallas)	H		+	+
**Family PORITIDAE**				
Alveopora verrilliana Dana	J,Y	+	+	+
A. fenestrata (Lamarck)			+	
A. spongiosa Dana			+	
Goniopora lobata Edwards & Haime		+	+	
G. djiboutensis Vaughan		+	+	+

Table 1. (continued).

Species	Previous record	Depth A	Depth B	Depth C
G. minor Crossland		+	+	+
G. columna Dana			+	+
G. stuchburyi Wells			+	
G. tenella (Quelch)	J,Y	+	+	
Porites (Porites) australiensis Vaughan	J,Y	+	+	+
P. (P.) solida (Forskal)		+	+	
P. (P.) murrayensis Vaughan		+	+	
P. (P.) lichen Dana	J,Y	+	+	
P. (P.) lobata Dana	J,Y	+	+	+
P. (P.) lutea Edwards & Haime	J,Y	+	+	+
P. (P.) cylindrica Dana	N		+	+
P. (P.) nigrescens Dana	K,Y		+	
P. (P.) tenuis Verrill	J,Y	+	+	
P. (P.) annae Crossland		+	+	
P. (P.) stephensoni Crossland		+	+	
P. (P.) compressa Dana	J,Y	+	+	+
P. (P.) cocosensis Wells *	J,Y			
P. (Synaraea) rus (Forskal)	N	+	+	
P. (P.) sp.				
Family FAVIIDAE				
Cyphastrea chalcidicum (Forskal)	M,K,J,Y	+	+	
C. microphthalma (Lamarck)	J,Y	+	+	
C. serailia (Forskal)		+	+	
Caulastrea furcata Dana			+	+
Diploastrea heliopora (Lamarck)	K,J,Y		+	+
Echinipora lamellosa (Esper)	K,J,Y		+	+
Favia favus (Forskal)	J,Y	+	+	+
F. pallida (Dana)	J,Y	+	+	+
F. rotumana (Gardiner)	J,Y	+	+	+
F. speciosa (Dana)	S,M,K,J,Y	+	+	+
F. stelligera (Dana)	J,Y	+	+	+
F. laxa (Klunzinger)	Y	+	+	
F. maxima Veron, Pichon & Wijsman-Best			+	+
F. maritima Nemenzo			+	
Favites abdita (Ellis & Solander)	M,K,J,Y	+	+	+
F. chinensis (Verrill)	N	+	+	+
F. complanata (Ehrenberg)	K,Y		+	
F. rotundata Veron, Pichon & Wijsman-Best			+	+
F. flexuosa (Dana)	J,Y	+	+	+
F. russelli (Wells)		+	+	
F. pentagona (Esper)	M,J,Y	+	+	+

Table 1. (continued).

Species	Previous record	Depth A	B	C
F. halicora (Ehrenberg)	J,Y	+	+	
Barabattoia amicorum (Edwars & Haime)	N		+	
Montastrea valenciennesi (E. & H.)	N	+	+	
M. curta (Dana)		+	+	
Goniastrea australiensis (E. & H.)	N	+	+	+
G. edwardsi Chevalier	N	+	+	+
G. aspera (Verrill)	N	+	+	+
G. pectinata (Ehrenberg)	M,K,J,Y	+	+	+
G. retiformis (Lamarck)	K,J,Y	+	+	+
Hydnophora exesa (Pallas)	J,Y	+	+	+
H. microconos (Lamarck)	K	+	+	
H. rigida (Dana)	J,Y		+	+
Leptoria phrygia (Ellis & Solander)	M,K,J,Y	+	+	+
Oulophyllia crispa (Lamarck)	M,J,Y		+	+
Platygyra pini Chevalier		+	+	+
P. lamellina (Ehrenberg)	M,K,J,Y	+	+	+
P. daedalea (Ellis & Solander)	N	+	+	+
P. sinensis (Edwards & Haime)	N	+	+	+
Plesiastrea versipora (Lamarck)	M,K,J,Y	+	+	+
Leptastrea purpurea (Dana)	M,K,J,Y	+	+	+
L. pruinosa Crossland	J,Y	+	+	
L. transversa Klunzinger		+	+	
Family OCULINIDAE				
Galaxea fascicularis (Linnaeus)	K,J,Y	+	+	
G. cf. astreata (Lamarck)		+	+	
Simplastrea versicularis Umbgrove	J,Y		+	
Family MERULINIDAE				
Merulina ampliata (Ellis & Solander)	K,J,Y	+	+	+
Scapophyllia cylindrica (E. & H.)	J,Y		+	+
Family PECTINIIDAE				
Echinophyllia asper (Ellis & Solander)	K,J,Y		+	+
Oxypora lacera (Verrill)			+	+
O. glabra Nemenzo			+	+

Table 1. (continued).

Species	Previous record	Depth A	Depth B	Depth C
Mycedium elephantotus (Pallas)	N		+	+
Pectinia lactuca (Pallas)			+	+
P. paeonia (Dana)			+	+
Family **MUSSIDAE**				
Blastomussa sp.			+	
Scolymia cf. vitiensis Bruggemann				+
Acanthastrea echinata (Dana)	M,K,J,Y	+	+	+
A. hillai Wells		+	+	
Lobophyllia hemprichii (Ehrenberg)	M,K,Y		+	+
L. corymbosa (Forskal)	K,J,Y		+	+
L. hataii Yabe, Sugiyama & Eguchi			+	
Symphyllia recta (Dana)	M,K,J,Y	+	+	+
S. radians Edwards & Haime			+	+
S. agaricia Edwards & Haime	M,K,J,Y		+	+
S. cf. valenciennesii Edwards & Haime			+	+
Family **CARYOPHYLLIIDAE**				
Euphyllia (E.) glabrescens C. & E.	K,J,Y	+	+	
E. (E.) cristata Chevalier			+	
E. (Fimbryaphyllia) ancora V. & P.	N	+	+	
Plerogyra sinuosa (Dana)			+	+
Physogyra lichtensteini (E. & H.)			+	+
Family **DENDROPHYLLIIDAE**				
Turbinaria peltata (Esper)	K,J,Y		+	+
T. frondens (Dana)	N		+	+
T. mesenterina (Lamarck)	N		+	+
T. reniformis Bernard			+	+
T. immersa Yabe & Sugiyama	J,Y		+	+
Tubastrea aurea Quoy & Gaimard	J,Y		+	+
Dendrophyllia micranthus (Ehrenberg)				+
**SUBCLASS OCTOCORALLIA**				
**ORDER COENOTHECALIA**				
Family **HELIOPORIDAE**				
Heliopora coerulea (Pallas)	K,J,Y	+	+	+

Table 1. (continued).

Species	Previous record	Depth A	B	C
ORDER STOLONIFERA				
Family TUBIPORIDAE				
Tubipora musica Linnaeus	K,J,Y	+	+	
CLASS HYDROZOA				
ORDER MILLEPORINA				
Family MILLEPORIDAE				
Millepora platyphylla H. & E.	K,J,Y,R	+	+	
M. dichotoma Forskal	R	+	+	
M. tenera Boschma	K,J,Y,R	+	+	
M. murrayi Quelch	K,J,R	+	+	
M. intricata Edwards & Haime	J,Y,R	+	+	
M. foveolata Crossland	R	+	+	
M. tuberosa Boschma	R	+	+	

Table 2. A list of alcyonacean corals known from the fringing reefs of Southern Taiwan. '*' indicates the species reported by Utinomi (1959). Distribution of coral species is based on collection records and field observation records from the following depths, A: recorded from 0-5 m; B: recorded from 5-15 m; C: recorded from 15-25 m.

species	Depth		
	A	B	C
CLASS ANTHOZOA			
SUBCLASS OCTOCORALLIA			
ORDER ALCYONACEA			
Family ALCYONIIDAE			
Alcyonium simplex Thomson and Dean	+	+	
A. molle Thomson and Dean	+	+	
A. rotundum Thomson and Dean	+	+	
Sinularia densa (Whitelegge)	+	+	+
S. exilis Tixier-Durivault	+	+	
S. facile Tixier-Durivault	+	+	
S. flexibilis (Quoy & Gaimard)		+	+
S. gibberosa Tixier-Durivault		+	+
S. grandilobata Verseveldt		+	+
S. granosa Tixier-Durivault	+	+	+
S. halversoni Verseveldt		+	
S. inexplicita Tixier-Durivault		+	
S. lochmodes Kolonko		+	+
S. mayi Lüttschwager *		+	
S. mollis Kolonko		+	
S. muralis May		+	+
S. numerosa Tixier-Durivault	+	+	+
S. polydactyla (Ehrenberg) *	+	+	+
S. scabra Tixier-Durivault	+	+	+
S. sp. 1		+	
S. sp. 2		+	
Lobophytum batarum Moser	+	+	
L. crassum Von Marenzeller	+	+	+
L. mirabile Tixier-Durivault		+	+
L. pauciflorum (Ehrenberg)	+	+	+
L. sarcophytoides Moser		+	+
L. solidum Tixier-Durivault		+	
L. sp.		+	
Sarcophyton crassocaule Moser	+	+	+
S. ehrenbergi Von Marenzeller *		+	+
S. glaucum (Quoy & Gaimard)	+	+	+
S. stellatum Kükenthal		+	
S. tortuosum Tixier-Durivault		+	
S. trocheliophorum Von Marenzeller *	+	+	+
S. sp. 1		+	

Table 2. (continued)

species	Depth		
	A	B	C
S. sp. 2		+	
Cladiella sphaerophora (Ehrenberg) *		+	
C. pachyclados (Klunzinger) *		+	
C. sp.		+	
Family NEPHTHEIDAE			
Nephthea erecta Kükenthal *		+	+
N. chabroli Audouin		+	+
Paralemnalia thyrsoides (Ehrenberg) *	+		
Family XENIIDAE			
Anthelia formosana Utinomi *	+	+	
Heteroxenia elisabethae Kölliker *	+	+	
H. sp.		+	
Cespitularia stolonifera Gohar *	+		
Family ASTEROSPICULARIIDAE			
Asterospicularia laurae Utinomi	+		

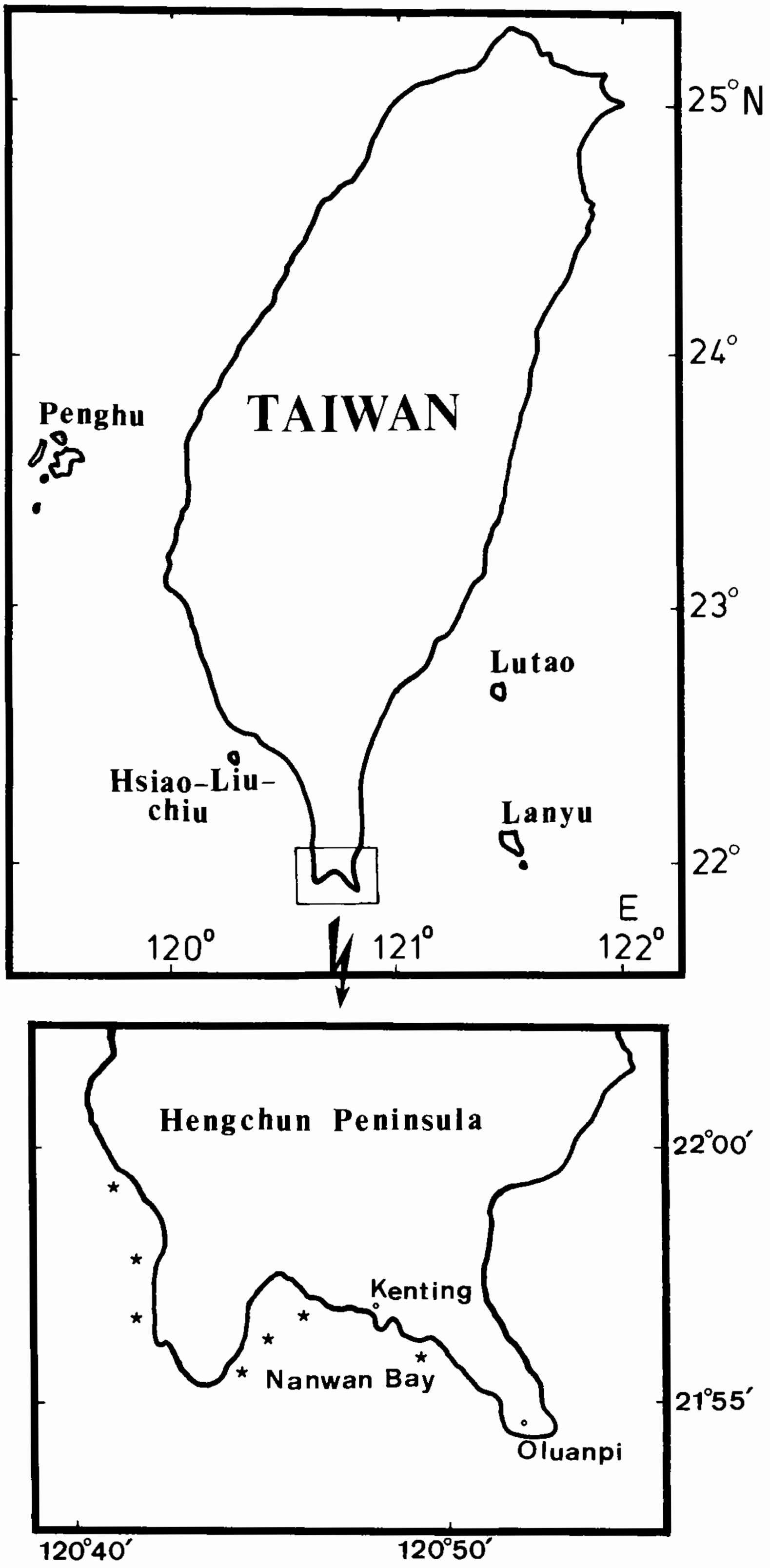

Fig. 1. Map of Taiwan and the study area. "*" indicates the study sites.

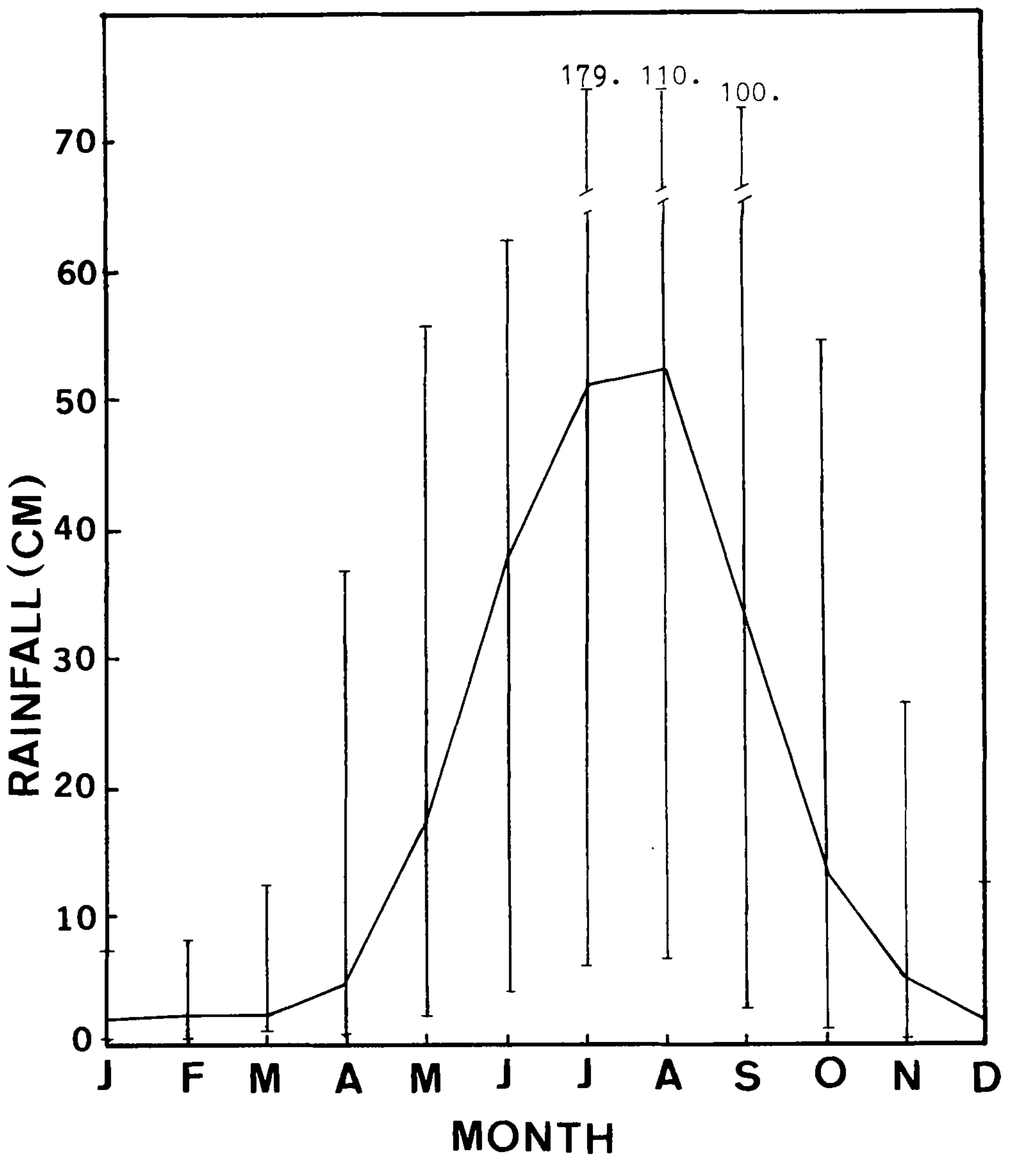

Fig. 2. Seasonal variation of monthly mean rainfall from 1900 to 1985 in southern Taiwan. Ranges are shown in bars. (Data from Hengchun Weather Station, Central Weather Bureau, R. O. C.)

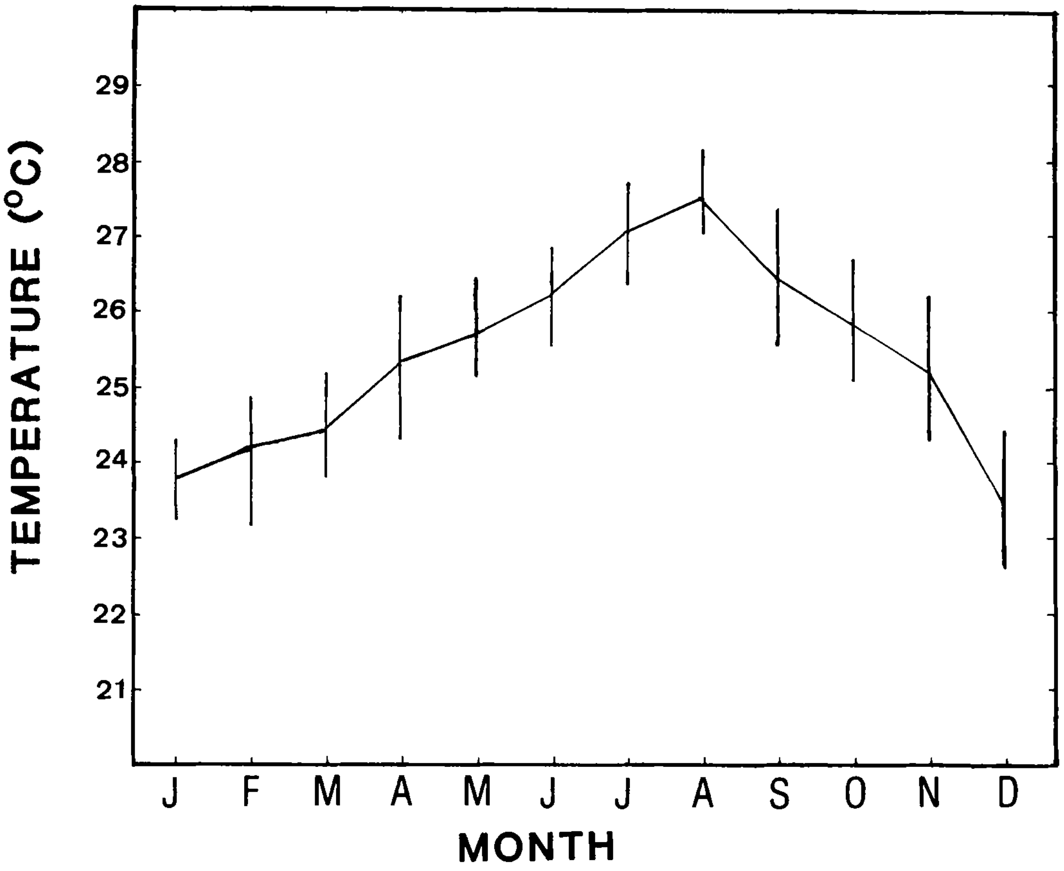

Fig. 3. Seasonal variation of monthly mean sea temperature in 15 m deep in Nanwan Bay from 1980-1988. (Data from the Radiation Laboratory, Taiwan Power Company, R. O. C.).

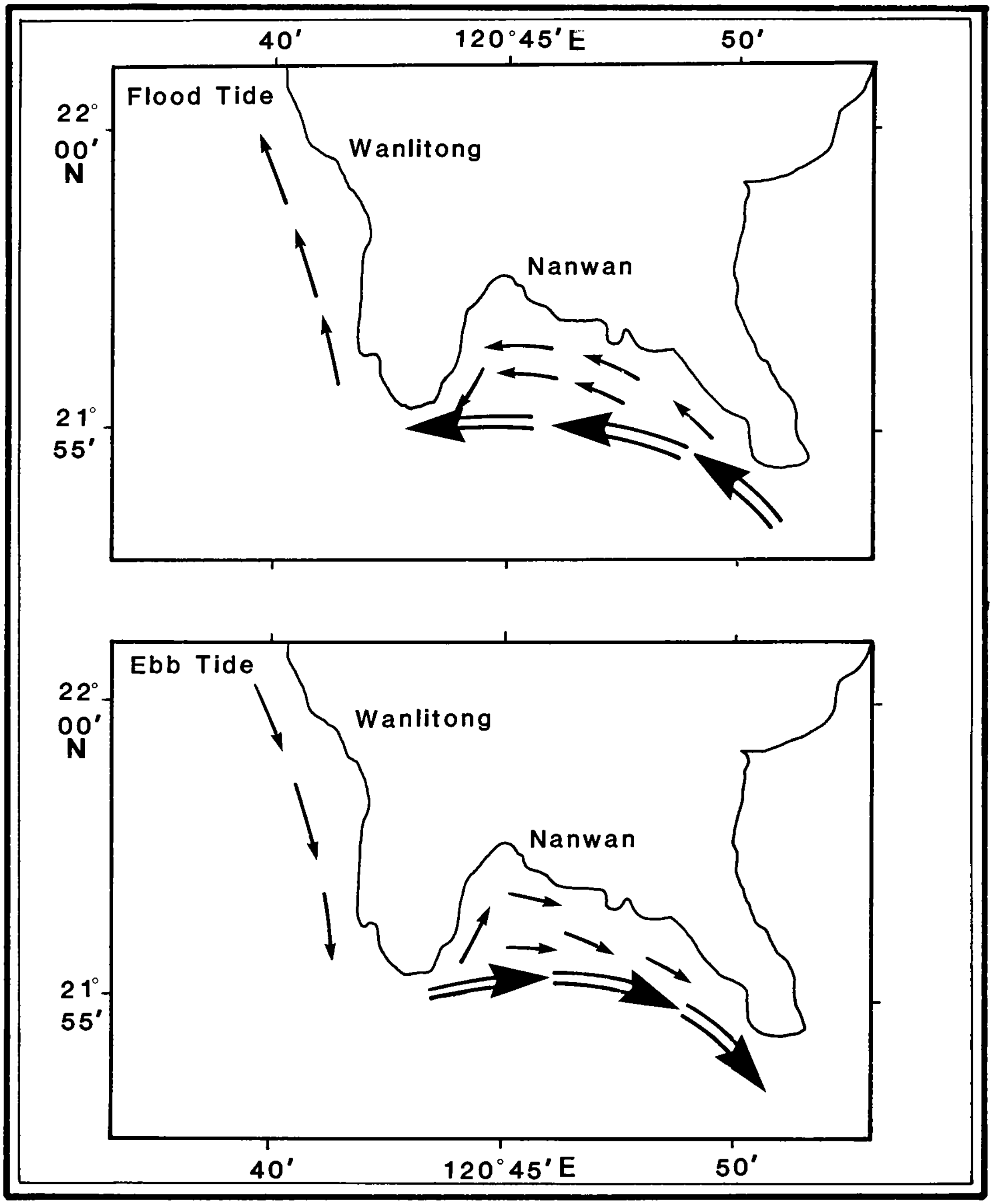

Fig. 4. Flow pattern of coastal waters in southern Taiwan during flood and ebb tides. (Redrawn from Hung et al. 1984 and Chang and Chen, 1987).
