# Supplementary figures and images for: Can resistant coral-Symbiodinium associations enable coral communities to survive climate change? A study of a site exposed to long-term hot water input
Source: PeerJ. 2014 Apr 8;2:e327. doi: 10.7717/peerj.327 (PMC3994648; doi:10.7717/peerj.327)

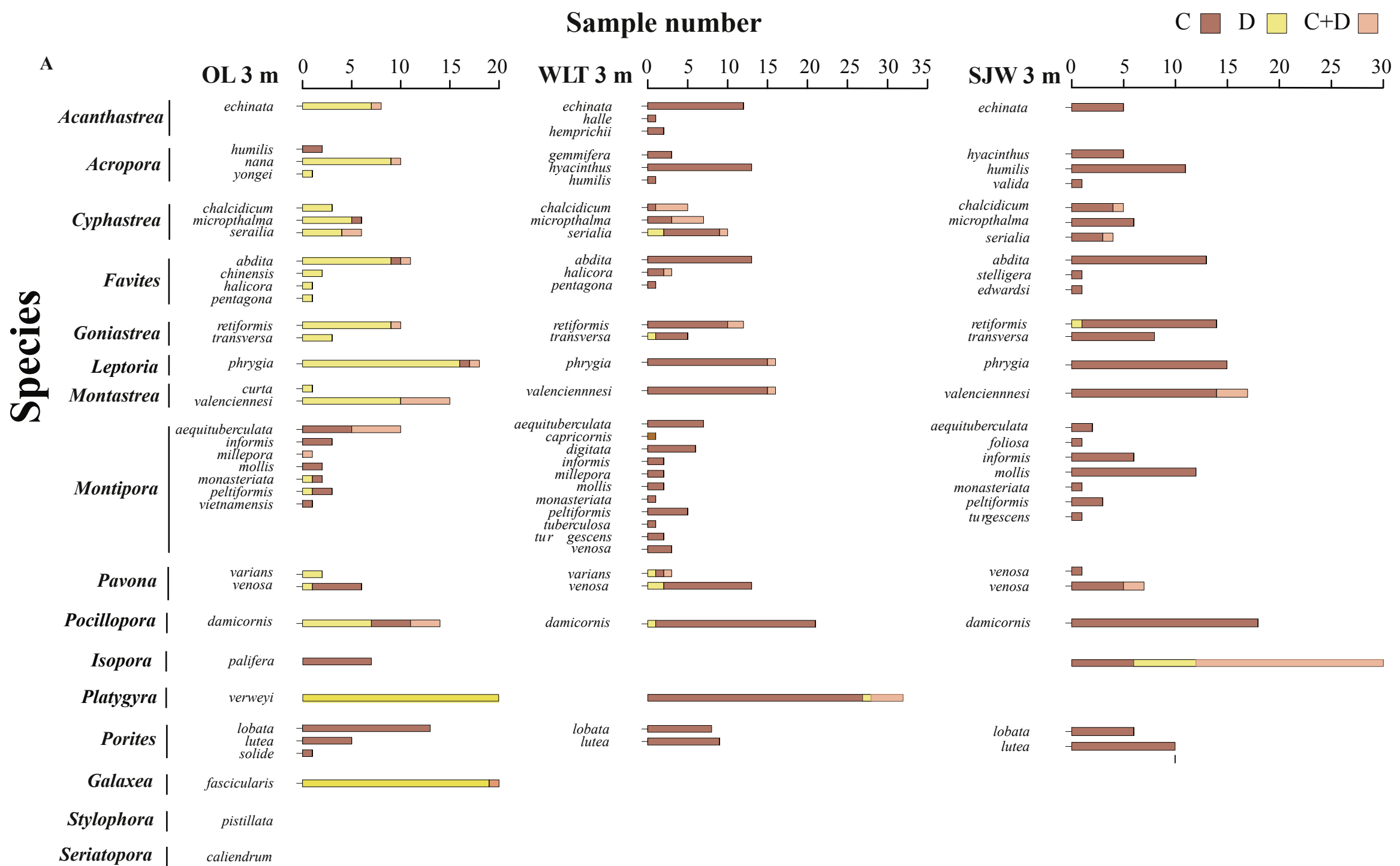

Figure S1

Supplement: Figure S1A — Brown bars, Symbiodinium C; light brown bars Symbiodnium C + D, and yellow bars, Symbiodnium D. [file peerj-02-327-s003.pdf]

## Sample number

C D C+D

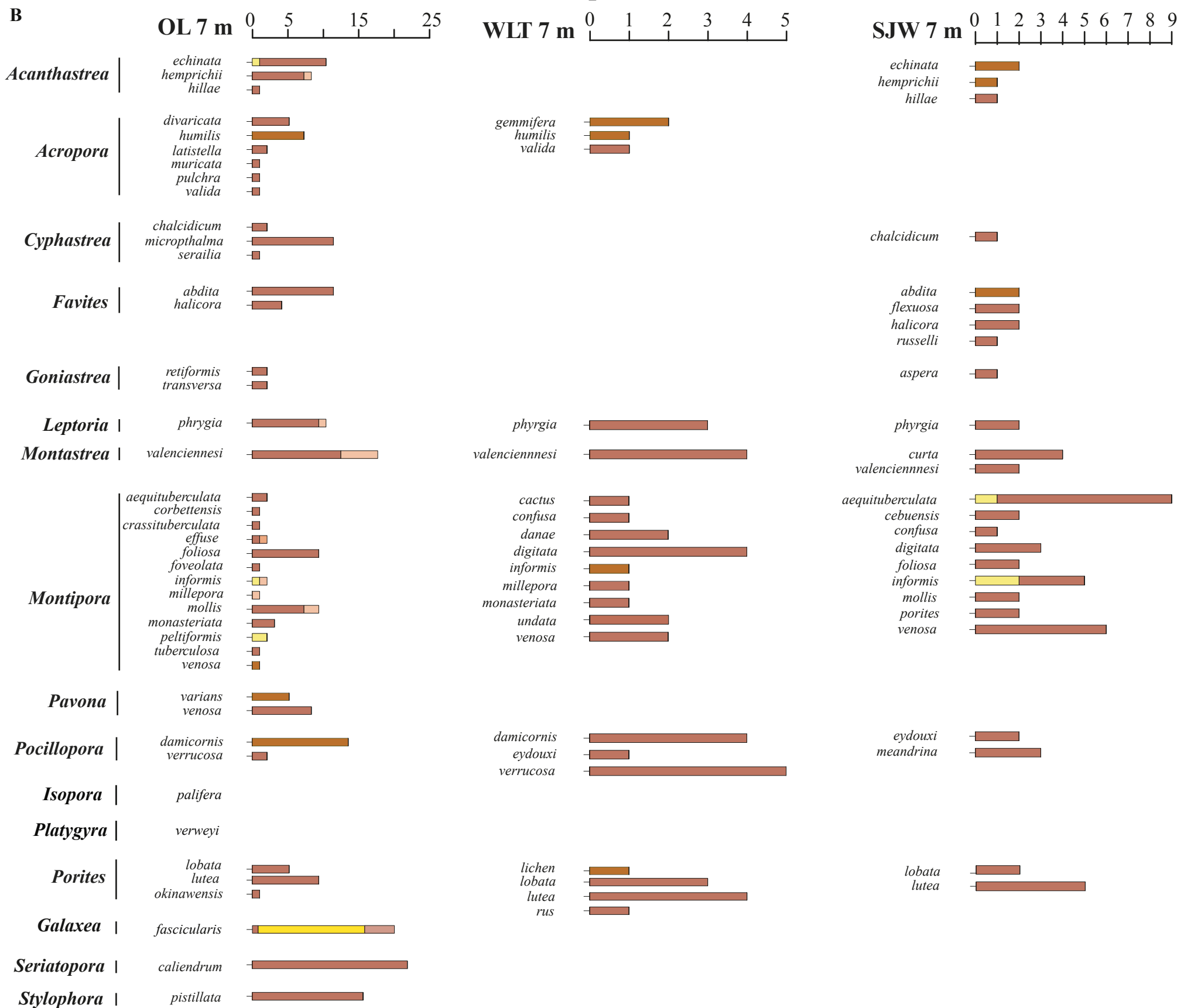

Figure S1

Supplement: Figure S1B — Brown bars, Symbiodinium C; light brown bars Symbiodnium C + D, and yellow bars, Symbiodnium D. [file peerj-02-327-s004.pdf]
